# The role of intramural combat martial arts in enhancing well-being among international students: a combined theoretical approach

**DOI:** 10.3389/fpsyt.2025.1582731

**Published:** 2025-04-11

**Authors:** Young-Taek Oh, Min-Ah Ryu, Jun-Phil Uhm

**Affiliations:** ^1^ Department of Kinesiology, Jeju University, Jeju, Republic of Korea; ^2^ Department of Sport Science, Jeonbuk National University, Jeonju, Republic of Korea; ^3^ Department of Kinesiology, Inha University, Incheon, Republic of Korea

**Keywords:** intramural martial arts, international students, positive emotion, stress, ego-resilience, well-being, self-determination theory, broaden-and-build theory

## Abstract

**Introduction:**

International students often face significant psychological challenges as they navigate new cultural, academic, and social environments. Drawing on Self-Determination Theory and the Broaden-and-Build Theory, this study aimed to examine how engagement in combat martial arts enhances well-being among international students through the sequential processes of positive emotions, stress relief, and ego-resilience.

**Method:**

A total of 311 international college students who had participated in intramural combat martial arts activities were recruited through an online survey platform. Serial mediation modeling was conducted to evaluate the hypothesized model using SPSS PROCESS.

**Results:**

Our results indicate that while engagement in combat martial arts positively influences well-being, its direct effect on ego-resilience was not significant. Positive emotions and stress relief independently mediated the relationship between physical activity engagement and well-being. The findings revealed a sequential mediation effect, where positive emotions, stress relief, and ego-resilience collectively mediated the relationshipbetween physical activity engagement and well-being.

**Discussion:**

This study contributes to the existing literature by providing insights into the psychological mechanisms underlying international students’ well-being, and offers important practical implications for promoting mental health and resilience among this population.

## 1 Introduction

The mental health of international students has long been a concern for researchers and practitioners. A recent study by Gao et al. (1), which surveyed over 400 international students from universities in 65 countries, found that more than half reported experiencing mental health challenges related to social isolation, cultural adjustment, and academic stress. These findings align with previous research that has similarly highlighted mental health concerns among international student populations worldwide (2, 3). International students, as a minority in unfamiliar settings, frequently encounter substantial obstacles stemming from language barriers, social isolation, and the absence of established support networks (4). Previous research has shown that long-term exposure to these stressors increases the risk of developing mental health disorders such as anxiety and depression (e.g., 5, 6). Given the growing number of international students worldwide, it is important to intervene to prevent adverse effects on student well-being and academic achievement (7). Accordingly, universities should support international students’ adjustment and mental health protection (6).

Participating in physical activities such as intramural combat martial arts may be an effective strategy to promote students’ well-being and support their adjustment (8). Unlike casual physical activities (e.g., jogging), combat martial arts provide a structured, goal-oriented environment, such as progressing through belt rankings, where participants can gain both physical and psychological benefits (9–11). Martial arts help strengthen coping mechanisms by alleviating stress and promoting discipline and emotional regulation through physical activity (12). In addition to stress relief and emotional regulation, martial arts have also been shown to reduce and channel aggressive tendencies, which may play a significant role in promoting psychological well-being. A meta-analysis by Harwood et al. (13) demonstrated that structured martial arts programs were associated with reductions in aggressive behavior, particularly in youth populations. This suggests that the self-regulatory demands and disciplined environment of martial arts may help individuals manage emotional impulses more effectively. For international students coping with stress and adjustment challenges, this capacity for emotional regulation through controlled physical expression can contribute meaningfully to overall mental well-being (14). Although the general benefits of physical activity on well-being are well known, research investigating the unique mechanisms by which combat martial arts consistently improves mental health in international students is limited. Understanding these mechanisms is essential for developing targeted evidence-based strategies in university settings.

Combat martial arts, such as judo, karate, and taekwondo, emphasize discipline, physical exertion, and skill mastery while also serving as an effective means of stress relief. Beyond their physical benefits, these martial arts cultivate core psychological attributes, including emotional regulation (15). For international students, who often navigate the challenges of being a minority in an unfamiliar cultural environment, martial arts provide not only physical engagement but also opportunities for social interaction, fostering connections with local customs and a sense of belonging (10). That is, combat martial arts can facilitate meaningful interpersonal interactions that help students adapt to cultural differences while building social networks. These experiences might mitigate external stressors and contribute to overall well-being (16). However, the psychological mechanisms that link stress relief, ego-resilience, and sustained well-being remain underexplored, limiting the effectiveness of current intervention strategies.

Psychological frameworks are the key to comprehending how combat martial arts foster short-term stress alleviation and long-term psychological benefits. Self-Determination Theory (SDT) emphasizes the role of combat in martial arts might promote ego-resilience and well-being by meeting three basic psychological needs: autonomy, competence, and relatedness (17). Autonomy is fulfilled when students make independent choices and set personal goals during training. Competence develops as students master techniques and progressively improve their physical and mental performance. Relatedness arises from social ties established with instructors and peers, fostering a supportive training environment. These satisfying experiences foster intrinsic motivation, leading to positive emotional states and sustained improvements in well-being (17). Similarly, the enjoyment derived from martial arts practice plays a key role in sustaining motivation and psychological benefits. For example, Ciaccioni et al. (18) demonstrated how older adult judo practitioners experienced increased intrinsic motivation and emotional engagement through enjoyable training experiences. Enjoyment may enhance the satisfaction of basic psychological needs and reinforce the likelihood of continued participation, which is crucial for long-term mental health benefits. However, while SDT is frequently utilized in physical activity research (e.g., 19), it often does not address how the aggregation of positive experiences during physical activity contributes to long-term outcomes, such as psychological resilience.

Broaden-and-Build Theory can complement SDT by explaining how positive emotional experiences lead to long-term adaptive resources. Positive emotions such as joy, satisfaction, and accomplishment from combat training broaden students’ thought-action repertoires and encourage greater psychological flexibility and stress management (20). These positive emotional experiences can facilitate initial stress relief, which, when experienced consistently, accumulates and enhances ego-resilience, which is defined as the capacity to adjust to adversity while preserving emotional stability (21, 22). International students frequently face academic, cultural, and social challenges; thus, developing ego resilience is crucial for navigating hardships and maintaining well-being. Integrating SDT with the Broaden-and-Build Theory may provide a comprehensive framework for connecting short-term emotional relief to long-term psychological development.

Based on SDT and Broaden-and-Build Theory, the current study aims to examine how engagement in combat martial arts enhances well-being among international students through the sequential processes of positive emotions, stress relief and ego-resilience. Specifically, it investigated how combat martial arts satisfy basic psychological needs, generate positive emotional experiences, and lead to long-term adaptive outcomes. By addressing both immediate and sustained improvements in mental health, this study informs the development of tailored interventions that promote the successful adaptation and integration of international students in university settings.

## 2 Theoretical background and hypothesis development

### 2.1 Self-determination theory and the effects of physical activity

Physical activity has long been recognized as a key contributor to well-being, with benefits extending beyond physical fitness, including improvements in mood, energy, life satisfaction, and psychological functioning (23). SDT (17) provides a practical framework for understanding why participation in physical activities, such as combat martial arts, can lead to ego-resilience and long-term well-being. This theory emphasizes the role of intrinsic motivation—participation driven by internal rewards such as personal satisfaction and growth—in promoting sustained engagement and positive outcomes. Combat martial arts are uniquely suited for fostering intrinsic motivation. They combine physical challenges with goal setting, skill mastery, and social interaction to create a comprehensive experience that supports holistic development (24).

Combat martial arts involve a progressive structure in which participants continuously work toward mastering new techniques and achieving personal milestones (25). The incremental nature of these achievements promotes ongoing self-improvement that builds self-confidence and enhances life satisfaction (17). For example, successfully learning a complex technique or earning a new belt offers participants a tangible sense of accomplishment, reinforcing their intrinsic motivation and commitment to training (26). This sustained engagement leads to cumulative benefits, such as improved mood regulation and an enhanced sense of purpose (27). Studies have shown that individuals engaged in structured physical activities with goal-oriented progression experience greater psychological benefits than those participating in less structured forms of exercise (28).

Participation in combat martial arts can foster long-term well-being by promoting personal resilience and a positive outlook on self-development (28). The repetitive cycle of setting goals, overcoming challenges, and achieving mastery instills persistence and discipline, essential for maintaining well-being (29, 30). Unlike activities that offer immediate gratification, combat martial arts emphasize delayed rewards through gradual progress and skill refinement, encouraging participants to remain committed and develop resilience over time (26). This process enhances physical fitness and promotes psychological growth as individuals develop a stronger belief in their ability to overcome obstacles and achieve future goals (31, 32). Accumulating positive experiences strengthens ego-resilience and enhances overall well-being, leading to lasting improvements in life satisfaction and emotional stability.

By integrating physical engagement with cognitive and emotional development, combating martial arts may provide an optimal environment for fostering ego-resilience and promoting sustained well-being (28). Participants experience immediate benefits, such as improved mood and energy, and long-term adaptive outcomes, including ego-resilience and well-being (8, 33). Combat martial arts represent a holistic approach to well-being, combining the benefits of physical exertion with personal growth and self-development. Based on this theoretical foundation and empirical support, we hypothesize the following:

H1-1: Engagement in physical activity in combat martial arts positively affects well-being.
H1-2: Physical activity engagement in combat martial arts positively affects ego-resilience.

### 2.2 Broaden-and-Build Theory and the mediating roles of positive emotion and stress relief

The Broaden-and-Build Theory (20) highlights the critical role of positive emotions in expanding individuals’ thought-action repertoires, which, in turn, helps them adopt new coping strategies, engage in flexible thinking, and develop long-term psychological resources. Positive emotional experiences such as joy, satisfaction, and accomplishment broaden cognitive and behavioral responses, encouraging individuals to seek adaptive behaviors that promote sustained well-being (20). Similarly, participation in combat martial arts can foster positive emotions through goal achievement, technique mastery, and social interactions. For example, successfully learning a difficult technique or progressing through belt ranks generates a sense of competence and emotional fulfillment (26). These positive emotions increase self-confidence, improve mood, and promote sustained engagement in health-promoting activities.

Stress relief is another key mechanism that links physical activity engagement to well-being. Combat martial arts promote stress relief through both physiological and psychological pathways. Physiologically, the high-intensity physical exertion of martial arts training triggers the release of endorphins, which are known for their mood-enhancing and stress-reducing effects (8). Additionally, physical activity moderates the body’s cortisol levels, helping mitigate the physical symptoms of chronic stress, such as muscle tension and fatigue (34). Psychologically, the structured nature of martial arts training shifts the participants’ focus to the present moment, promoting mindfulness and mental clarity (35). Participants focus on task-specific goals, such as executing precise movements or refining techniques, allowing them to temporarily detach themselves from external stressors (36).

Stress relief through combat martial arts can benefit international students, who often encounter elevated stress due to academic pressure and cultural adjustment challenges (37). Regular engagement in training provides an outlet for releasing accumulated tension while promoting relaxation and emotional regulation. As participants experience repeated stress relief, they gain increased mental clarity, which enhances their ability to focus on academic tasks and social interactions and fosters overall well-being (12). Empirical evidence has shown that consistent physical activity coupled with mental engagement leads to sustained improvements in life satisfaction, particularly in individuals facing chronic stress (38).

Stress relief offers immediate psychological benefits and fosters an environment in which positive psychological outcomes accumulate, enhancing well-being over time. By reducing the intensity of negative emotional states, such as anxiety and worry, individuals can experience more frequent positive emotions, reinforcing adaptive behaviors (20). For example, when international students feel less overwhelmed by external stressors, they become more open to social engagement, academic challenges, and personal growth. The cumulative effects of reduced stress and broadened cognitive responses promote sustainable well-being, creating a positive feedback loop in which continued participation in physical activity fosters emotional and psychological benefits. Based on this, we propose the following hypothesis:

H2-1: Positive emotions mediate the relationship between engagement in physical activity and well-being.
H2-2: Stress relief mediates the relationship between physical activity engagement and well-being.

### 2.3 Broaden-and-Build Theory and the mediating role of ego-resilience

The Broaden-and-Build Theory (20) emphasizes that positive emotions are not only momentary states but also key contributors to the development of long-term psychological resources, such as ego-resilience. As individuals accumulate positive emotional experiences through regular participation in combat martial arts, they develop enhanced psychological flexibility and adaptive capacity, allowing them to recover quickly from setbacks and navigate adversity (39). Combat martial arts provide participants with frequent opportunities to face and overcome challenges, whether by mastering complex techniques or competing in sparring matches. Each success reinforces feelings of accomplishment, persistence, and self-discipline, which cumulatively contribute to the development of ego-resilience (40).

Ego-resilience plays a central role in promoting sustained well-being by enabling individuals to maintain emotional stability and effectively adapt to new and challenging situations (41). For international students, who frequently encounter stressors such as language barriers, academic demands, and unfamiliar social environments, ego-resilience can serve as a vital resource for managing uncertainty and overcoming difficulties. Combat martial arts foster this resilience by instilling persistence and teaching students to embrace challenges as opportunities for growth rather than as obstacles (12). This mindset helps students maintain an optimistic outlook and view adversity as a stepping stone toward future achievements, thereby supporting long-term well-being (42). The cumulative impact of these positive coping strategies ensures that international students can navigate cultural and academic challenges with greater confidence and psychological stability.

In addition to its direct effects of positive emotions, stress relief serves as a catalyst for the development of ego-resilience and well-being. The reduction of stress through physical engagement in martial arts frees cognitive and emotional resources, allowing individuals to shift their focus toward goal-directed behaviors, such as skill mastery and achievement (35). As individuals experience repeated cycles of stress relief and success, they adopt proactive coping strategies that enhance their adaptability and reinforce their resilience (40). This dynamic interaction between stress relief and ego-resilience underscores their interconnected role in fostering long-term well-being (43). When individuals experience sustained stress relief and increased resilience, they are more likely to sustain positive emotional states, engage meaningfully in daily life, and derive satisfaction from their accomplishments (20, 41).

Ego-resilience can be critical in sustaining well-being because it not only equips individuals to manage current stress but also improves their ability to face future challenges with emotional stability and optimism (44). By developing resilience through combat martial arts, participants may create a strong psychological foundation that leads to long-term life satisfaction, improved emotional regulation, and an enhanced sense of purpose. For international students, this resilience can translate to better academic performance, stronger social integration, and overall positive life outcomes. Based on this theoretical framework, we hypothesized:

H3-1: Ego-resilience positively mediates the relationship between engagement in physical activity and well-being.
H3-2: Positive emotions, stress relief, and ego resilience sequentially mediate the relationship between engagement in physical activity and well-being.

## 3 Materials and methods

### 3.1 Participants

Data were collected through an online survey administered via Qualtrics and distributed on Reddit.com. Reddit is a widely used online community platform with a large and diverse user base, while Qualtrics is a well-established tool commonly used for creating and distributing online surveys in academic research. Both platforms have been frequently adopted in previous studies targeting diverse or hard-to-reach populations. Data collection took place from April to June 2024. After obtaining approval from subreddit moderators, the survey link was posted in communities related to international students and traditional martial arts. Participation was entirely voluntary, and individuals who were interested in the study were invited to complete the questionnaire via Qualtrics. The present study received ethical approval from the Institutional Review Board (IRB) of the authors’ affiliated university.

A total of 420 responses were collected; however, 109 incomplete responses were excluded, resulting in a final sample of 311 international college students. Participants were eligible for inclusion if they had been actively practicing combat martial arts for at least one year. Look more closely in to participants, among the respondents, 180 (57.878%) were male, and 131 (42.122%) were female. The average age of participants was 21.44 years (*SD* = 1.11), ranging from 19 to 23 years. The majority of participants were from Asian countries (190; 61.093%), followed by the Middle East (75; 24.116%), and Europe (46; 14.790%). All participants were engaged in intramural combat martial arts activities at their respective universities, with 102 (32.798%) participating in Taekwondo, 76 (24.437%) in Judo, 72 (23.151%) in Karate, 40 (12.862%) in Jiu-Jitsu, and 21 (6.752%) in other combat martial arts. Regarding training experience, 183 participants (58.842%) had been training combat martial arts for at least one year, 86 participants (27.653%) for two years, and 42 participants (13.505%) for more than three years. Also, the majority of participants trained at least once per week. More specifically, participants reported training between one and five times per week, with an average training frequency of approximately 1.741 sessions per week.

### 3.2 Measures

We adapted items from existing studies that demonstrated adequate reliability and validity. The selected items were revised and reworded for the combat martial arts context based on suggestions from experts —including a high-level Taekwondo practitioner (6th dan), a post-doctoral researcher, and a sport management professor—to ensure content relevance and item clarity. Specifically, 15 items from the Sport Engagement Scale (SES; 45) were used to measure physical activity engagement. 14 items from the Ego-Resiliency Scale (ER89), developed by Block and Kremen (21), were adopted to assess ego-resiliency. Positive emotions were measured using 10 items from the PANAS scale (46). 10 items from the Perceived Stress Scale (PSS-10; 47) were used to assess the extent to which international students perceived stress in daily life. The scores on perceived stress were reverse-coded to assess stress relief, following the approach used by Yu and Kim (48), which applied reverse coding to measure reductions in perceived stress levels. Lastly, well-being was assessed using eight items from the Psychological Well-Being Scale (49). All scales were measured using a 5-point scale.

### 3.3 Statistics and data analysis

**Figure 1:**
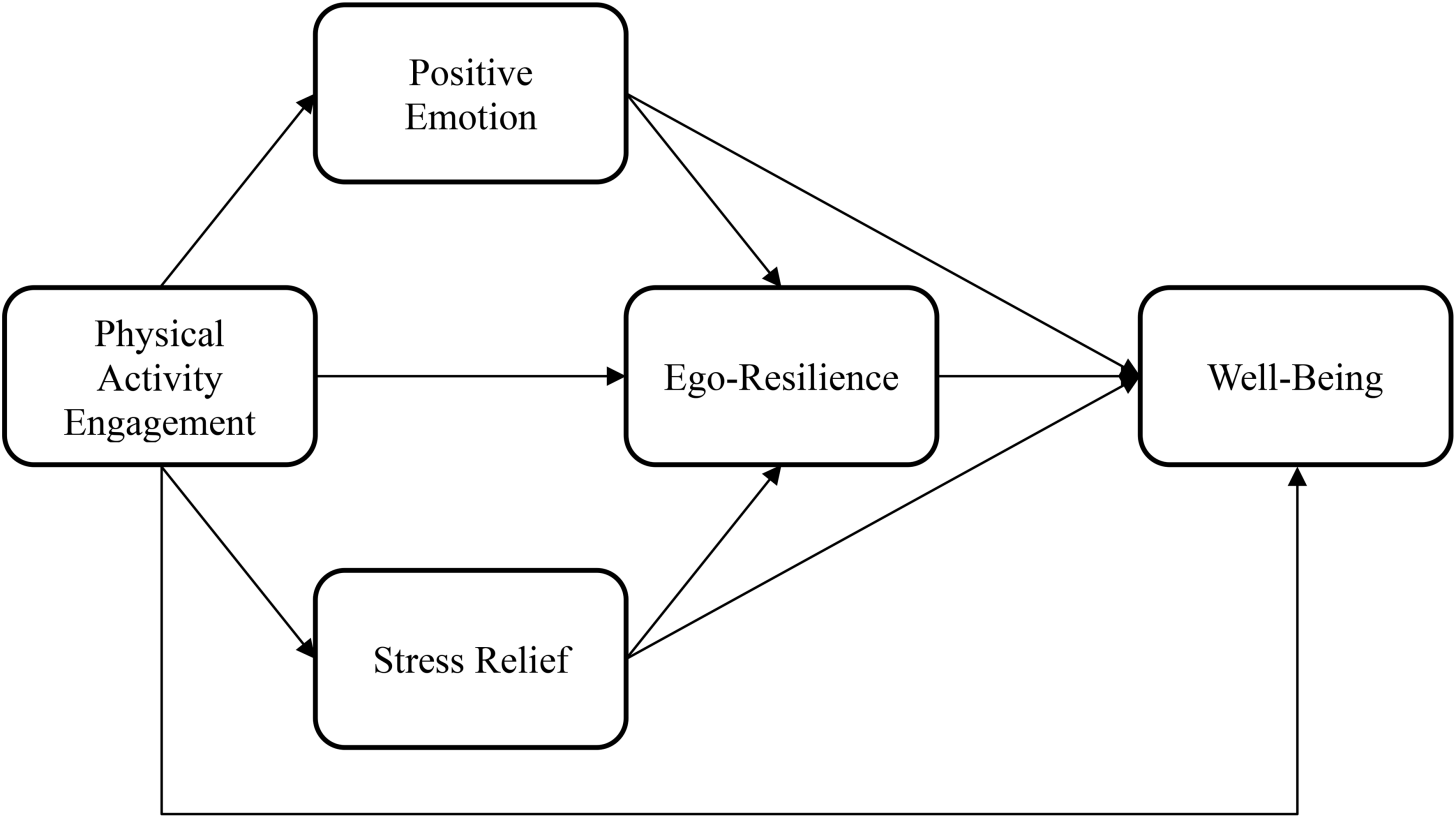
Research model.

The authors screened the collected data and checked its basic assumptions prior to further analysis. Descriptive statistics were analyzed to assess the normality of the data distribution. To examine multicollinearity, correlation analysis was conducted. Internal consistency was assessed using Cronbach’s alpha. To test our hypotheses, we developed a serial mediation model and assessed it using the PROCESS macro in SPSS (Model 6; 50). In accordance with the proposed research model, we set physical activity engagement in combat martial arts as the independent variable, and well-being as the dependent variable. Positive emotion, stress relief, and ego-resilience were incorporated as the first, second, and third mediators, respectively (see **Figure 1**).

### 3.4 Descriptive statistics and correlation

Following the recommendations from Hair et al. (51), the data demonstrated a normal distribution, as the skewness and kurtosis values were within the recommended ranges of -2 to +2 and -7 to +7, respectively. We performed a correlation analysis to check for multicollinearity issues in our research model. As all the correlation coefficients were less than.85 and the range of the variance inflation factor value was from 1.225 to 2.739, which were less than the threshold of 10 (51), we confirmed that there was no multicollinearity issue. The correlation matrix and descriptive statistics of the items are reported in **Table 1**.

**Table 1:** Descriptive and correlational statistics of research variables.

Variable	1	2	3	4	5
1. Physical activity engagement	--				
2. Positive emotion	.413**	--			
3. Stress relief	.386**	.766**	--		
4. Ego-resilience	.270**	.555**	.511**	--	
5. Well-Being	.405**	.630**	.582**	.786**	--
*M*	3.593	3.367	3.313	3.567	3.624
*SD*	.635	.518	.529	.577	.651
Skewness	-.044	.650	.764	-.462	-.661
Kurtosis	-.472	-.140	.120	1.010	.862
Cronbach’s *α*	.804	.876	.882	.850	.879

**p<.01.

### 3.5 Common method bias

To mitigate concerns regarding potential common method bias in the survey design, Harman’s one-factor test was conducted through an exploratory principal component factor analysis of all measurement items. According to Podsakoff et al. (52), common method bias is considered a concern when a single factor accounts for more than 50% of the total variance. The results indicated that the largest single factor explained only 26.89% of the variance. Since no single factor accounted for the majority of the covariance among the independent and dependent variables, common method bias was not deemed a significant issue in this study.

## 4 Results

The path coefficient estimates, including confidence intervals for the model, are reported in **Table 2**. The results showed that while the direct effect of physical activity engagement in combat martial arts on well-being was statistically significant (*b* = .120, *SE* = .046, 95% *CI* = [.029,.211]), supporting Hypothesis 1-1, the direct effect on ego-resilience was not statistically significant (*b* = .029, *SE* = .041, 95% *CI* = [-.052,.109]), leading to the rejection of Hypothesis 1-2. Hypotheses 2-1 and 2-2 were supported, as the indirect effects through positive emotion (*b* = .086, *SE* = .023, 95% *CI* = [.044,.139]) and stress relief (*b* = .009, *SE* = .005, 95% *CI* = [.001,.022]) were statistically significant. Furthermore, while the indirect effect of ego-resilience on the relationship between physical activity engagement and well-being was not significant (*b* = .020, *SE* = .032, 95% *CI* = [-.041,.087]), leading to the rejection of Hypothesis 3-1, the indirect effect of physical activity engagement on well-being through positive emotion, stress relief, and ego-resilience was significant (*b* = .039, *SE* = .012, 95% *CI* = [.018,.064]), supporting Hypothesis 3-2.

**Table 2:** The results of direct and indirect effects.

	Effect	*SE*	95% *CI*	
			LL*CI*	UL*CI*
*Direct Effect*				
DV: Positive Emotion				
Physical activity engagement	.337***	.037	.305	.787
DV: Stress Relief				
Physical activity engagement	.070*	.029	.013	.127
Positive Emotion	.747***	.035	.678	.817
DV: Ego-Resilience				
Physical activity engagement	.029	.041	-.052	.109
Positive Emotion	.430***	.072	.289	.571
Stress Relief	.223*	.069	.086	.358
DV: Well-Being				
Physical activity engagement	.120*	.046	.029	.211
Positive Emotion	.135*	.055	.027	.242
Stress Relief	.695	.039	.619	.772
Ego-Resilience	.254***	.059	.139	.369
*Indirect Effect*				
Through Positive Emotion (M1)	.086*	.023	.044	.139
Through Stress Relief (M2)	.009*	.005	.001	.022
Through Ego-Resilience (M3)	.020	.032	-.041	.087
Through M1, M2, and M3	.039*	.012	.018	.064

*p<.05; ***p<.001; DV, dependent variable; LL*CI*, lower level of confidence interval; UL*CI*, upper level of confidence interval.

## 5 Discussion

### 5.1 General discussion

The results of testing Hypothesis 1-1 showed a statistically significant direct effect of physical activity engagement in combat martial arts on well-being. This finding aligns with the SDT (17), which argues that psychological well-being can be enhanced when individuals engage in activities that meet the basic psychological needs for autonomy, competence, and relatedness. Numerous studies have shown that structured physical activity, particularly skill-based and goal-directed exercises, can contribute to well-being by fostering a sense of mastery and self-efficacy (e.g., 53). Engaging in combat martial arts offers a systematic framework for gradual skill advancement, reinforcing a sense of competence and personal achievement (26). Additionally, the social context of combat martial arts training, which encompasses peer and instructor interactions, can cultivate relatedness, a crucial element of intrinsic motivation that promotes psychological well-being (54).

A plausible explanation for the results of Hypothesis 1-1 is that engaging in combating martial arts offers an optimal balance between structured challenges and autonomous goal setting, both of which are critical for sustained psychological well-being (25). Unlike other physical activities that primarily emphasize free play or competition, martial arts emphasize progressive goal attainment through belt-ranking systems, technical mastery, and self-regulation, which may contribute uniquely to long-term engagement and psychological growth (26). Previous studies have indicated that individuals who engage in structured physical activities requiring self-discipline and continuous skill refinement tend to experience higher self-esteem, self-regulation, and well-being (28). These findings suggest that the positive impact of physical activity engagement on well-being is not merely due to physical movement but can also be driven by the fulfillment of basic psychological needs embedded in structured training environments.

The rejection of Hypothesis 1-2, which proposes a direct effect of physical activity engagement in combat martial arts on ego-resilience, suggests that improvements in resilience may not result solely from participation but rather from additional psychological mechanisms. These findings align with the study by Son and Lee (55), which reported no significant difference in ego-resilience levels between individuals who participated in structured exercise and those who did not. Resilience is often understood as a dynamic process rather than a fixed trait (56), requiring individuals to develop adaptive coping strategies in response to stressors rather than merely facing physical challenges (57, 58). This aligns with research suggesting that resilience emerges through repeated exposure to manageable stressors and cognitive adaptation to challenges rather than an automatic outcome of physically demanding activities (59).

A key takeaway from the rejection of Hypothesis 1-2 is that martial arts engagement still fosters resilience through indirect pathways rather than direct effects. This study identified stress relief and positive emotional responses as mediators, indicating that resilience building is not solely a function of exposure to adversity but also how individuals emotionally and cognitively process their experiences. This underscores the importance of understanding the psychological mechanisms in sports engagement (60). Rather than diminishing the role of physical activity, our findings reinforce the multifaceted nature of its psychological impact, suggesting that additional mediators, as well as contextual factors, may further explain how combat martial arts participation contributes to psychological growth.

The support for Hypothesis 2-1, which found that positive emotions significantly mediated the relationship between physical activity engagement and well-being, aligns with the Broaden-and-Build Theory (20). This theory posits that positive emotions expand individuals’ cognitive and behavioral repertoires, allowing them to develop psychological resources that promote adaptive coping and resilience. Physical activity has long been recognized as a source of positive affect, and previous research has suggested that exercise-induced positive emotions contribute to enhanced mental well-being (61, 62). In martial arts training, positive emotional experiences such as enjoyment, pride, and self-confidence are particularly relevant as they encourage continued participation and contribute to long-term psychological benefits (63, 64).

Beyond the immediate emotional improvement, the psychological effects of positive emotions can accumulate over time, shaping individuals’ approaches to adversity and stress regulation (65). Positive emotional experiences during training can help individuals adopt proactive coping strategies, build psychological resiliency, and sustain motivation in facing challenges (66). Empirical studies have shown that individuals who regularly experience positive emotions are more likely to demonstrate greater resilience as these emotions contribute to cognitive reappraisal and adaptive behavioral responses to stress (67). These findings suggest that positive emotions are not merely fleeting experiences but play a crucial role in strengthening long-term psychological well-being, highlighting the importance of emotional engagement in martial arts participation.

The support for Hypothesis 2-2, which identified stress relief as a mediator between physical activity engagement and well-being, further validated the Broaden-and-Build Theory by illustrating how emotional regulation contributes to psychological resource building. Combat martial arts training involves intensive physical movements, strategic thinking, and controlled decision-making, all of which demand focus and mental discipline, temporarily diverting attention from external stressors (68, 69). This effect is similar to mindfulness practices, in which individuals become immersed in their physical and cognitive experiences, enhancing mental clarity and stress reduction (69). Research has consistently shown that regular engagement in physical activity is associated with lower cortisol levels and reduced psychological distress, reinforcing the idea that structured exercise is an effective stress regulation strategy (e.g., 70, 71).

Alongside the physiological benefits, stress relief from martial arts training fosters an adaptive emotional environment, enabling individuals to respond to challenges more effectively and develop a more balanced approach to adversity. A meta-analysis by Rebar et al. (72) found that structured exercise interventions significantly reduced the symptoms of stress, anxiety, and depression, with disciplined physical activities, such as martial arts, producing particularly strong effects. These findings align with the Broaden-and-Build Theory, which proposes that reducing stress allows individuals to broaden their cognitive and emotional capacities, ultimately making them more adaptable to future challenges (20). Given that martial arts training emphasizes discipline, controlled aggression, and self-regulation, it may serve as a particularly effective means of enhancing stress management capabilities, further supporting its role in promoting long-term well-being.

Our results indicate that Hypothesis 3-1 was rejected, while Hypothesis 3-2 was supported. Hypothesis 3-1 proposed that ego-resilience alone mediates the relationship between physical activity engagement in combat martial arts and well-being. In contrast, Hypothesis 3-2 posited that positive emotions, stress relief, and ego-resilience sequentially mediate this relationship. The rejection of Hypothesis 3-1 suggests that ego-resilience alone is insufficient to mediate the impact of physical activity on well-being. However, the support for Hypothesis 3-2 indicates that ego-resilience becomes a meaningful contributor to well-being when it follows the development of positive emotions and stress relief.

A possible explanation for these findings is that resilience requires a foundation of emotional stability and stress adaptation before it can meaningfully contribute to well-being. While resilience is often conceptualized as a trait that enables individuals to recover from setbacks and maintain emotional stability in the face of adversity, emerging research suggests that it is not merely an inherent characteristic but a dynamic process shaped by repeated exposure to positive emotional reinforcement and effective stress management strategies (73). Similarly, Cohn et al. (59) suggested that resilience develops over time as individuals accumulate experiences in emotional regulation, stress management, and cognitive adaptation. Without consistent exposure to positive emotions and effective stress-regulation strategies, individuals may struggle to develop the cognitive flexibility and psychological strength necessary for resilience. These findings reinforce the view that resilience is not an isolated or static trait but rather a dynamic process influenced by prior emotional experiences and coping mechanisms, which explains why it functions as a mediator only when preceded by positive emotional experiences and stress relief.

### 5.2 Theoretical and practical implications

Our study advances the understanding of how structured physical activity contributes to psychological well-being and resilience by integrating SDT (17) and the Broaden-and-Build Theory (20). The findings reinforce the idea that psychological benefits from physical activity extend beyond mere participation and are influenced by underlying psychological mechanisms. While need fulfillment in basic psychological needs plays a direct role in enhancing well-being, resilience appears to develop through a gradual process involving emotional regulation and stress adaptation. This distinction is significant, as it highlights different pathways through which structured physical activities contribute to long-term psychological outcomes. The current study supports the notion that positive emotional experiences and stress relief act as critical mechanisms through which individuals build psychological resources over time. Combat martial arts training, characterized by goal-directed progression, controlled exposure to adversity, and structured skill development, provides an environment where emotional and cognitive wellness are cultivated. These findings contribute to the broader theoretical discourse on how structured physical engagement in combat martial arts can foster long-term adaptability, challenging traditional views that assume resilience results solely from repeated exposure to physical challenges.

The findings offer valuable insights for universities aiming to enhance student well-being and resilience through structured physical activity programs. Given the psychological benefits observed in combat martial arts training, universities should consider integrating structured physical activities that emphasize skill mastery, emotional regulation, and goal progression into their wellness initiatives. To maximize these benefits, universities should design programs that explicitly incorporate psychological skill-building elements alongside physical training. Instructors should be trained to create an autonomy-supportive environment that fosters intrinsic motivation and emotional resilience. Additionally, structured self-reflection exercises and mindfulness techniques can be incorporated to help students process their experiences, reframe challenges, and enhance long-term psychological adaptation. Universities should also consider interdisciplinary collaborations between sports programs, mental health services, and academic departments to create holistic well-being interventions that integrate structured physical activity with psychological support. By emphasizing both the physical and psychological aspects of structured training, universities can create a more comprehensive and sustainable model for student well-being and personal development.

### 5.3 Limitations and suggestions

While the current study provides valuable insights into the psychological benefits of physical activity engagement in combat martial arts, several limitations should be acknowledged. First, the study employed a cross-sectional design, which limits the ability to infer causal relationships between martial arts participation and psychological outcomes. More specifically, while the findings suggest that structured physical activity contributes to well-being and resilience through positive emotions and stress regulation, the directionality of these relationships remains uncertain. Future research should utilize longitudinal designs to examine how martial arts training influences well-being and resilience over time, allowing for a more comprehensive understanding of its long-term psychological impact.

Another limitation of our study lies in the measurement of stress relief. To the best of our knowledge, there is no specific scale that directly measures stress relief; however, several indirect approaches can be considered, such as adaptive coping and resource accumulation. Future studies could incorporate measures that capture the dynamics of stress relief, including adaptive coping (e.g., Brief COPE Inventory; 74) and resource accumulation (e.g., Conservation of Resources Evaluation; 75). These tools may offer deeper insights into how individuals recover from or buffer stress through positive experiences. Such approaches can capture not only momentary relief but also long-term psychological resilience and overall well-being.

While previous studies have emphasized the importance of specific positive emotions such as enjoyment in understanding the psychological benefits of martial arts (e.g., 18), our study focused on measuring general positive emotional experiences rather than distinct emotional states. As a result, we did not include specific enjoyment measures such as the Physical Activity Enjoyment Scale (PACES), which has been widely used to assess enjoyment in physical activity contexts. Future research should incorporate such targeted instruments to examine how combat martial arts enhance enjoyment and, in turn, contribute to long-term well-being outcomes.

Lastly, while this study focused on positive psychological mechanisms such as stress relief and emotional regulation, other psychological factors may also play a role in the relationship between martial arts engagement and well-being. For instance, self-efficacy, cognitive appraisal of challenges, and personality traits may influence how individuals experience and respond to martial arts training (e.g., 76, 77). Future research should examine additional psychological mediators and moderators to provide a more nuanced understanding of the pathways through which structured physical activity fosters mental health benefits. By addressing these limitations, future studies can contribute to a more comprehensive and culturally inclusive understanding of the role of martial arts in promoting psychological well-being and resilience.

## Data Availability

The original contributions presented in the study are included in the article/supplementary material. Further inquiries can be directed to the corresponding author.
